# Assessing functional reorganization in visual cortex with simulated retinal lesions

**DOI:** 10.1007/s00429-021-02366-w

**Published:** 2021-09-16

**Authors:** Holly D. H. Brown, André D. Gouws, Richard J. W. Vernon, Samuel J. D. Lawrence, Gemma Donnelly, Lorraine Gill, Richard P. Gale, Heidi A. Baseler, Antony B. Morland

**Affiliations:** 1 Department of Psychology, University of York, York, UK; 2 York Neuroimaging Centre, University of York, York, UK; 3 York Biomedical Research Institute, University of York, York, UK; 4 Institute of Neuroscience and Psychology, University of Glasgow, Glasgow, UK; 5 Department of Health Sciences, University of York, York, UK; 6 Academic Unit of Ophthalmology, York Teaching Hospital NHS Foundation Trust, York, UK; 7 Hull York Medical School, University of York, York, UK

**Keywords:** Macular degeneration, Lesion projection zone, Functional reorganization, fMRI, Visual cortex, Feedback

## Abstract

Macular degeneration (MD) causes central vision loss, removing input to corresponding representations in the primary visual cortex. There is disagreement concerning whether the cortical regions deprived of input can remain responsive, and the source of reported cortical responses is still debated. To simulate MD in controls, normally sighted participants viewed a bright central disk to adapt the retina, creating a transient ‘retinal lesion’ during a functional MRI experiment. Participants viewed blocks of faces, scrambled faces and uniform grey stimuli, either passively or whilst performing a one-back task. To assess the impact of the simulated lesion, participants repeated the paradigm using a more conventional mean luminance simulated scotoma without adaptation. Our results suggest our attempt to create a more realistic simulation of a lesion did not impact on responses in the representation of the simulated lesion. While most participants showed no evidence of stimulus-driven activation within the lesion representation, a few individuals (22%) exhibited responses similar to a participant with juvenile MD who completed the same paradigm (without adaptation). Reliability analysis showed that responses in the representation of the lesion were generally consistent irrespective of whether positive or negative. We provide some evidence that peripheral visual stimulation can also produce responses in central representations in controls while performing a task. This suggests that the ‘signature of reorganization of visual processing’, is not found solely in patients with retinal lesions, consistent with the idea that activity may be driven by unmasked top–down feedback.

## Introduction

Macular degeneration (MD) is a progressive eye disease which causes the loss of vision in the central part of the visual field. Following degeneration of the retina, the corresponding representations in primary visual cortex (V1) are deprived of input. This has led to a number of studies exploring whether the region deprived of cortical input, known as the ‘lesion projection zone’ (LPZ), might reorganise and take on a new role, processing inputs from intact retina (Baker et al. 2005, 2008; Baseler et al. 2011; Dilks et al. 2009; Hoffmann et al. 2003; Sunness et al. 2004). Determining the source of any LPZ responses is important; if there is evidence of remapping, or a more permanent reorganization of visual processing, this could interfere with the success of visual restoration (Baseler et al. 2011; Morland 2015). Successful visual perception can only occur if the parts of the visual brain which previously processed input from the macula remain available, and capable of processing new incoming information. However, there is debate concerning the state of visual cortex following visual loss, primarily within the LPZ (for a review, see: Brown et al. 2016). The inconsistency within the literature is partly attributed to differences in how cortical reorganization is defined, but also by what mechanism it occurs (Morland 2015). Reorganization can refer to structural changes occurring in the brain following acquired or congenital eye disease, but more frequently, it refers to functional changes, whereby patterns of neural activation differ from those observed in healthy individuals. For this study, reorganization refers to patterns of activity within the LPZ that cannot be explained by (a) known properties of the visual system and (b) the absence of visual input (Engel et al. 2015; Morland 2015).

Irrespective of the way in which reorganization is defined, there is also considerable variation in the proportion of patients that exhibit fMRI responses in the LPZ; estimates vary from zero to approximately 50% (Morland 2015). Given this variability, we sought to determine whether this property is unique to patients, or whether similar response variability can also be found in a sample of individual sighted controls using a simulated LPZ.

A common approach to determining if reorganization occurs is to measure responses in the LPZ. In cases of congenital visual loss, e.g. rod achromats who have a small central scotoma from birth, there is evidence of remapping of visual inputs (Baseler et al. 2002). In cases of acquired vision loss, the results are mixed. Positive responses in the LPZ to stimuli presented to intact peripheral visual field are frequently taken as a signature of reorganization of visual processing in MD (Baker et al. 2005, 2008). However, the mechanism underlying LPZ responses remains unknown. Furthermore, some studies report an absence of any positive responses in the LPZ, suggesting cortical reorganization does not occur in MD (Baseler et al. 2011; Smirnakis et al. 2005; Sunness et al. 2004).

One interpretation is that the LPZ starts to process information from the intact peripheral visual field not previously represented by this region of cortex (Baker et al. 2005, 2008; Dilks et al. 2009,2014). An alternative view is that the signals observed in the LPZ reflect normal feedback to V1 from extra-striate areas, unmasked in partially blind individuals when engaged in a task (Masuda et al. 2008,2010). Further evidence for the role of feedback mechanisms is provided in sighted individuals; when naturalistic scenes are partially occluded, stimulus-related information can be decoded in the unstimulated visual cortex which represents the occluded visual field (Petro et al., 2014; Smith and Muckli 2010; Williams et al. 2008). It appears therefore that even in normally sighted controls, there is evidence of feedback in fMRI signals, although they are revealed in a consistent pattern of responses across voxels, rather than in a univariate increase in mean signal as found in patients.

Given that evidence of feedback to V1 can be found in controls, we asked whether it could be unmasked in univariate responses in V1. To answer this question, we used a more realistic simulation of a retinal lesion in normally sighted controls and measured their responses in V1’s representation of the simulated lesion, the ‘sLPZ’. Masuda and colleagues (2008) speculated that the sLPZ signals are not observed in controls because retinal aftereffects signalled the presence of a uniform grey region, the ‘simulated lesion’. Therefore, we simulated the loss of central vision physiologically, not by manipulating the viewed images, but instead by temporarily (and reversibly) compromising photoreceptor function in the macula in normally sighted individuals.

Adaptation to high light levels can render targets at lower light levels undetectable for relatively long periods of time. The level of bleaching of the photo-pigment in outer segments of the cone photoreceptors accounts for this effect—higher light levels bleach the pigments more making the cones less sensitive to light (for a review see Barlow (1972). We therefore had participants adapt to a bright white disk, saturating signals from the cones and ensuring the simulated ‘lesion’ was in retinal coordinates, irrespective of eye movements much like a genuine scotoma. We also selected a luminance for our stimuli that made them undetectable within the adapted region. We reasoned that by making visual stimuli that were presented in the periphery undetectable in the adapted region, we might unmask influences of cortical feedback on the mean signal measured in this simulated sLPZ. We hypothesised that under conditions of bright light adaptation of the macular, we would detect signals in the sLPZ, which would be absent when controls viewed stimuli without prior adaptation. We also compared signals in the sLPZ during stimulus-related task and passive viewing conditions, predicting that signals would be more likely to emerge under the stimulus-related task condition as found previously in patients (Masuda et al. 2008).

Finally, we asked whether responses in the sLPZ (controls) and LPZ (patient) were consistent within individuals, or simply spurious or due to random chance. To do this, we repeated our fMRI acquisitions for each condition, and for each participant assessed the average univariate response and its reliability across repeated acquisitions. These data allowed us to gauge whether the responses we obtained from a patient with longstanding loss of macula vision were similar in scale and reliability to those we obtained from controls.

## Methods

### Participants

**Fig. 1:**
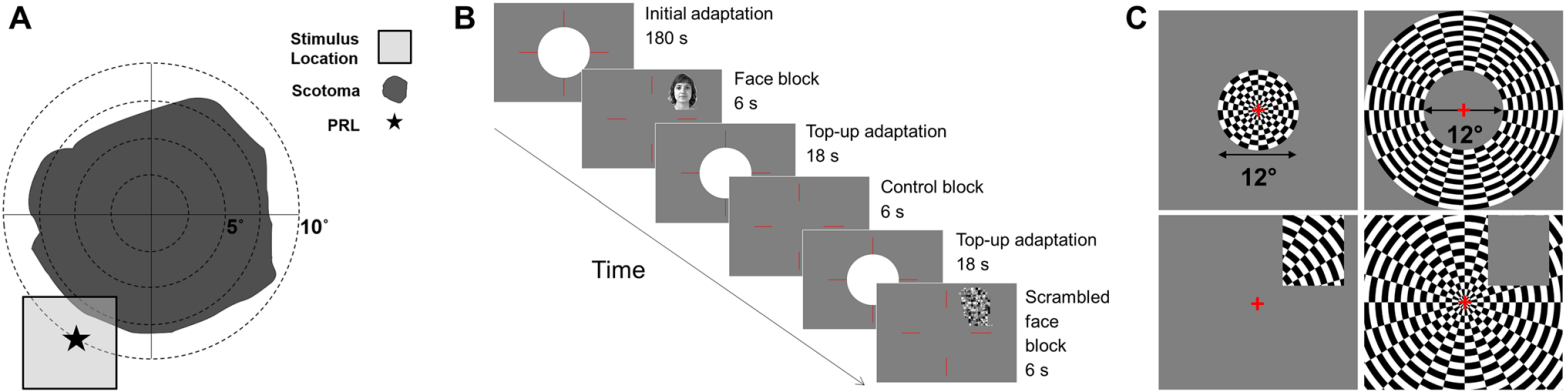
**a** Schematic of JMD’s central scotoma in the right eye. Preferred retinal locus (PRL) located in the lower left visual field, so stimuli were positioned here. The patient’s scotoma was absolute, meaning no stimulus could be detected within the defined region. Only the right eye was tested in the functional MRI experiment. **b** Schematic of main functional experiment (controls). Each run began with 180 s adaptation period; participants fixated centrally, using the red lines as a guide. 12 test blocks (6 s, comprising of either faces, scrambled faces or uniform grey) were presented, interleaved with top-up adaptation blocks (18 s) to ensure adaptation was maintained. Each stimulus (faces or scrambled faces) was presented in the upper right quadrant of the visual field for 800 ms, ISI of 200 ms. For the first 2 runs, participants passively viewed the stimuli. For the last 2 runs, participants completed a one-back task. Participants were given a response box and were asked to indicate when an image was repeated by pressing a button. Each functional run lasted 7 min 48 s. For illustrative purposes, the RGB values are not the same as used in the experiment as images were too dark and very low contrast. **c** Illustration of the sLPZ localiser (for controls only) and the stimulus localiser (for controls only)

Eleven participants were recruited for the current study; 10 normally sighted controls (4 males; ages 23–37, average age = 26.8 years) and one participant with Juvenile Macular Degeneration (Referred to as JMD throughout, male, aged 40 years). JMD was diagnosed with Stargardt’s disease, an inherited progressive disease, at 17 years old. JMD has bilateral absolute central scotomata (approximately 18 × 20 degrees of visual angle) and uses a preferred retinal locus (PRL) in the lower left visual field (fixating using an upper right retinal location at the edge of the scotoma). A visual representation of the scotoma, PRL and location of the stimulus is shown in Fig. 1A. All participants participated in fMRI experiments, but JMD also completed an additional behavioural experiment and visual assessments at York Teaching Hospital. Written consent was obtained for all participants and the study was approved by the York Neuroimaging Centre ethics committee in accordance with the Declaration of Helsinki*.*

### Imaging parameters

Scanning was performed at the University of York Neuroimaging Centre using a GE 3 Tesla HDx Excite MRI scanner.

#### Structural MRI

One high-resolution 3D T1-weighted, Fast Spoiled Gradient Echo pulse sequence (FSPGR) anatomical image was acquired (TR = 7.8 ms, TE = 2.9 ms, TI = 450 ms, voxel size = 1.13 × 1.13 × 1 mm^3^, flip angle = 20°, matrix size 256 × 256 × 176, FOV = 290 mm) using the 8-channel whole head High-Resolution Brain Array coil. Three T1-weighted anatomical images were acquired for each participant (TR = 7.8 ms, TE = 3.0 ms, TI = 600 ms, voxel size = 1 × 1 × 1 mm^3^, flip angle = 12°, matrix size 256 × 256 × 176, FOV = 256 mm) using a 16-channel Posterior Brain Array coil. One T2*-weighted fast gradient recalled echo scan was acquired (TR = 400 ms, TE = 4.3 ms, voxel size = 2 × 2 × 1 mm^3^, flip angle = 25°, matrix size 128 × 128, FOV = 260 mm) using the 16-channel coil. Finally, an axial proton density scan was also acquired during the functional MRI experimental session using the 16-channel coil and the same slice prescription to aid alignment between functional and high-resolution structural data (TR = 2700 ms, TE = 34.84 ms, voxel size = 1 × 1 × 2 mm, flip angle = 90, matrix size 192 × 192 × 39, FOV = 192 mm).

#### Functional MRI

All functional data were acquired on the 16-channel coil to improve the signal-to-noise in the occipital lobe using the following parameters: TR = 3000 ms, TE = 30 ms, voxel size = 2 × 2 × 2 mm^3^, flip angle = 90°, matrix size 96 × 96 × 39, FOV = 192 mm. Data were acquired using a slice prescription with coverage including occipital and temporal lobes.

### Stimulus generation

Stimuli were presented using a ProPixx LED projector (VPixx Technologies, Saint-Bruno, CA) and were rear-projected onto a textured screen in the bore of the MRI scanner, which could be viewed on the screen via a mirror attached to the head coil. To monitor participants’ fixation stability, we used an eye tracker to record a video of the eye, allowing us to monitor eye movements in real time throughout the experiment (https://www.crsltd.com/tools-for-functional-imaging/mr-safe-eye-tracking-for-fmri/livetrack-fmri/).

PNG images of faces were processed in MATLAB (Mathworks). First, all images were converted to grayscale with flattened histograms, to control grayscale ranges across images. Following this, the mean luminance value of each image was set to 705 cdm^−2^ so that they would be undetectable in regions of the retina that were adapted to bright light of ~15,000 cdm^−2^. All images were re-scaled to take up 50% of a 400 × 400-pixel size stimulus. Finally, all images were placed on a dark grey background matching mean luminance of the images. To scramble the faces, each image was divided into a 20 × 20 square grid and each square was randomly shuffled through ±90 or 180. Finally, we applied a slight blurring to the intact and the scrambled images with a Gaussian filter (SD = 1px) to soften the lines created when scrambled.

#### JMD behavioural test

We devised a short behavioural experiment to confirm the location of JMD’s PRL. Stimuli (mean size was ~ 7 × 9° of visual angle) comprised grayscale faces, scrambled faces, objects and control trials containing no stimuli. Stimuli were presented for 800 ms, appearing in any quadrant of the visual field, or in the centre of the screen. JMD was given an auditory cue when a stimulus was presented and was asked the following: (1) Did you see anything? (2) If yes, where was the stimulus? (3) Can you describe what the stimulus was? There were 40 trials (10 for each stimulus condition).

#### High light level adaptation experiment

Our aim was to use a bright adaptation stimulus to render the retina insensitive to visual stimuli that were presented. Previous work allowed us to estimate the level needed and to justify safe recovery after bleaching from 15,000 cdm^−2^ which was the maximum we could achieve with our set up (Czeh et al. 1965; Stockman and Sharpe 2006). To determine an adaptation regimen to render the stimuli undetectable, we measured detection thresholds for the stimuli within the adapted region. We found, after manipulating an initial and top-up period of adaptation, that stimuli at 705 cdm^−2^ were never detected following the three-minute adaptation and the following the subsequent top-up periods of 18 s. Red fixation lines were centred vertically and horizontally, occupying 20 of visual angle, to aid central fixation in the absence of an explicit central fixation marker (Fig. 1B). During the ‘adaptation’ period, the bright white disk (12° diameter, 15,000 cdm^−2^) was overlaid on the fixation lines. In control blocks, the circle changed to uniform dark grey (mean luminance: 705 cdm^−2^) to match the background and the luminance of stimuli (e.g., faces). For all functional runs, the size of the adaptation circle, red fixation lines, and position of the stimulus remained the same. The size of individual face and scrambled stimuli varied slightly; the mean size was ~ 7 × 9° of visual angle. Images were positioned 10° of visual angle from the centre of the screen to the centre of the image. For controls, stimuli were presented in the upper right quadrant of the visual field, abutting the edge of the adapted circle (Fig. 1B). For JMD, they were presented in the lower left quadrant, corresponding with the PRL. Position differed due to JMD being recruited after the control participants.

#### Retinotopic mapping

The travelling-wave method was used to acquire retinotopic maps to locate V1 in control participants (Engel et al. 1997). Stimuli were presented binocularly and consisted of high contrast (> 98%, mean luminance = 400 cdm^−2^) expanding checkerboard rings and 90° wedges rotating anti-clockwise. All checkerboards reversed at a rate of 6 Hz. Stimuli were presented on a grey background (200 cdm^−2^) with a red central fixation cross, traversing a circular region of radius 14.34°. Each cycle lasted 36 s, with 8 cycles per run (Vernon et al. 2016).

#### LPZ and stimulus localiser

For controls, a central radial checkerboard (12 diameter, reversal rate = 6 Hz) was presented for 12 s, followed by a peripheral checkerboard annulus (radius extending from 6 to 15 radius) also presented for 12 s (Fig. 1C, upper panel). This cycle was repeated 8 times to localise the representation of the simulated lesion. Using a similar paradigm, we used checkerboard stimuli to localise the region representing the stimulus (Fig. 1C, lower panel). We isolated the region where the stimuli were presented (upper right quadrant) and alternated between a flickering checkerboard occupying this region and the surrounding region. For JMD, the same paradigm was used, however, instead of a central radial checkerboard, a full field checkerboard alternated with uniform grey (mean luminance = 200 cdm^−2^). For all participants, stimuli were presented monocularly to the dominant eye only. Dominant eye was determined by having participants point to a corner of a room, then close each eye in turn and state during which interval their finger was closest to the corner.

### Experimental design

Control participants completed three scanning sessions: one structural and two functional sessions, which included sLPZ/stimulus localiser scans and main functional scans (one with and one without the ‘adaptation’ disk). JMD completed two sessions: one structural and one functional session including LPZ localiser and main functional experiment without adaptation.

#### MRI protocol for lesion simulations and control conditions

All functional scans were completed under monocular viewing conditions, testing the dominant eye only. For each experiment (adaptation and no-adaptation), all participants completed four functional runs; two passive viewing and two runs during which participants completed a one-back task. For the one-back tasks, participants were instructed to press a button when an image matched the previous one. For experiment 1, which included the ‘adaptation’ disk, each of the 4 functional runs were preceded by a 180 s adaptation period, followed by 12 stimulus blocks (4 blocks of each stimulus category: faces, scrambled faces and uniform grey control blocks, block duration = 6 s) interleaved with 11 top-up adaptation blocks (18 s). See Fig. 1B for further details. Stimulus blocks were presented in a pseudorandom order whereby each functional run had blocks presented in a different order, but the same four order sequences were presented to each participant. Six stimuli were presented per block (presented for 800 ms, with 200 ms inter-stimulus interval (ISI)). 18 s (uniform grey) was added at the end of the scan to allow us to capture the full hemodynamic response to the last stimulus. Participants were instructed to fixate centrally, where they perceived the red fixation lines to intersect. For experiment 2, the same procedures were used, but the initial adaptation period was reduced to 18 s and the white central disk was changed to uniform grey. This experiment served as a control condition, replicating the conventional simulated lesions used in previous research (Baker et al. 2005, 2008; Baseler et al. 2011; Masuda et al. 2008) to contrast with our new approach that attempted to simulate a retinal lesion with adaptation. Top-up adaptation intervals were replaced with a uniform grey disk to keep the timings of the functional runs consistent with experiment 1.

JMD also completed 4 functional runs consistent with experiment 2, the ‘no adaptation’ paradigm. Given JMD has a PRL in the lower left visual field, the stimuli were positioned in the lower left quadrant. The red fixation lines were expanded to be the full width/height of the screen to guide fixation and help JMD position their eyes centrally on the screen.

### Analysis

#### Anatomical data

Three high-resolution T1 isotropic images were aligned and then averaged. This average was then divided by the T2*-weighted data to correct for the gradient inhomogeneity caused by the 16-channel half head coil, and to improve the grey–white contrast. FreeSurfer software (http://surfer.nmr.mgh.harvard.edu/) was used to perform automatic segmentation of the averaged T1 scan. All automated segmentations were checked for quality and correctness and manual corrections were then performed where necessary to remove handles and bridges and fix missegmented white matter, using itkGray software (https://web.stanford.edu/group/vista/cgi-bin/wiki/index.php/ItkGray).

#### Retinotopic mapping

For our controls, we used the standard travelling-wave method to analyse our data using mrVista (http://web.stanford.edu/group/vista/cgi-bin/wiki/index/php/MrVista; Engel et al. 1997; Wandell et al. 2007). The first 3 volumes were removed to minimize the effects of magnetic saturation. Between- and within-scan motion correction was applied. Data were aligned to the high-resolution T1-weighted image, and to reduce noise we averaged across wedge scans and across ring scans (Vernon et al. 2016). An 8-cycle sine wave was applied to each voxel in turn; the phase with the best fit was assigned to each voxel. Retinotopic maps were viewed on inflated cortical surfaces which were constrained to grey matter. The region of interest (ROI) V1 was manually drawn on the partially inflated cortical surfaces derived from the grey/white brain segmentation created using ‘mrMesh’ (part of the mrVista software package). ROIs were drawn using mrVista, using phase reversals to identify boundaries. Retinotopic mapping was not performed on JMD.

#### LPZ localiser analysis

Individual participant fMRI data were analysed using FEAT (FMRI Expert Analysis Tool v4.1; Worsley 2001). The first 3 volumes were removed, the high-pass filter cut-off point was 100 s (correcting for low frequency drift), FILM pre-whitening and spatial smoothing (Gaussian kernel with 5 mm FWHM spatial smoothing) were used, and motion was corrected for (motion parameters were also entered as confound covariates). Stimuli were entered as an explanatory variable, convolved with a gamma hemodynamic response function (HRF), and contrasts were run to compare stimuli to baseline. This allowed us to isolate responses to the central 12° stimulus, and the surrounding annulus. We also included blink event files (derived from eye-tracker data) as additional confound variables as they can be an additional source of noise (Gouws et al. 2014). Data were cluster corrected (*Z* > 2.3, *p* < 0.05) and were registered to the high-resolution structural space, using the axial proton density scan to aid alignments.

#### High light level adaptation experiment fMRI analysis

Upon initial analysis, we identified artefacts in some participants (including JMD) around the sagittal sinus and took additional measures to remove them and applied these steps to all participants. Functional data were first motion corrected. We then took the cumulative sum of absolute differences (CSAD) across volumes within a functional run in each participant. We then converted these values to *Z* scores. We reasoned that as fluid flow within the sagittal sinus would elicit rapid changes across volumes leading to larger CSAD values, any voxels with a CSAD value greater than 3 standard deviations should be excluded (de Zwart et al. 2005; Olman et al. 2007).

The remaining first level analyses were the same as for the LPZ localiser. The first 60 volumes from the adaptation runs were removed as this constituted the initial adaptation period. For experiment 2 (no-adaptation condition), the first 6 volumes were removed. We used spatial smoothing (Gaussian kernel with 4 mm FWHM spatial smoothing) for both experiments. All three stimulus conditions were entered as separate explanatory variables (EVs), convolved with a double-gamma HRF, and two contrasts were run: faces versus control blocks, scrambled faces versus control blocks. This allowed us to isolate any possible effects of stimuli. Individual participant data were entered into a higher-level group analysis. The two task runs were combined, as were two passive runs for each participant using fixed-effects analysis with cluster correction (*z* > 2.3, *p* < 0.050).

#### ROI analysis

V1 ROIs for all control participants were restricted to central and peripheral representations, using LPZ localiser data. Each participant therefore had sLPZ ROI and surround (not used in subsequent analyses) in the hemisphere contralateral to the visual field where stimuli were presented. We also had a stimulus representation ROI for each control participant, derived in a similar manner using the stimulus localiser (see Fig. 1, lower panel in part C). Due to the poor quality LPZ localiser data in JMD, we drew an ROI in the cortical surface. We were careful to define the LPZ ROI to fall well within the projection of the retinal lesion and also of a comparable size and location of the sLPZ defined in controls. This ensured that our LPZ ROI would not capture BOLD signals at the stimulus representation. We were unable to make a principled selection of the ROI that would sensibly capture the BOLD response to the stimulus because the stimulus location in JMD differed from that presented to controls, because of the size and location of the visual field deficit.

In addition to the hand-drawn surface ROI, we also utilised the Benson atlas for JMD to determine if this would be a more suitable and more objective way of defining the LPZ in V1 (Benson et al. 2012, 2014). We also applied this to one example control participant so that we were able to directly compare the functionally defined ROI in the control against a retinotopic map generated by the Benson atlas. We found that the atlas overestimated the size of the LPZ ROI in both JMD and the example control. Whilst this did overlap with our original ROIs, the Benson atlas was twice the size in the control (in terms of number of voxels) despite specifying the central 6 only, and more than three times the size in JMD, including a greater proportion of the anterior calcarine sulcus, likely capturing the stimulus representation in both participants. Having identified the shortcomings of the atlas for this particular question concerning LPZ responses, we opted for our more conservative hand-drawn ROI for JMD.

All ROI analysis was performed at the individual level using FSL’s FEATquery. This was applied to the COPE (contrast of parameter estimates) statistics for each stimulus type, and subsequently converted into mean percent signal change. This gave us a measure of mean response to each stimulus type and task condition in each ROI.

#### Data visualisation

For each participant, a partially inflated surface derived from the grey/white brain segmentation was created using ‘mrMesh’ (part of the mrVista software package), and functional data (thresholded zstat images from FSL) were imported as parameter maps into mrVista to view on the inflated surface with ROIs.

#### Reliability analysis

To interrogate the reliability of responses across functional runs for each viewing condition, multivoxel pattern analysis was used to characterise the pattern of responses across voxels within the LPZ, which were then correlated across two runs using Pearson’s *R*. Values were subsequently converted into Fisher *Z* scores to ensure data were normally distributed. Each participant had four reliability scores: Faces with task, faces with passive viewing, scrambled faces with task, scrambled faces with passive viewing.

#### Classifier analysis

We used the two measures from our fMRI data, response amplitude and the reliability of the response, to classify responses into two categories—those associated with the unstimulated LPZ or the stimulus representation. We applied linear discriminant analysis with leave-one-out cross-validation to train the classifier on adaptation and no-adaptation control data separately. Once the classifier had been trained on all of the control data, we applied it to JMD’s data to establish whether the responses from their LPZ were best categorized as those originating from the LPZ or stimulus representation; exploring whether signals in JMD’s LPZ were stimulus-driven or not.

## Results

### Individual LPZ BOLD responses

**Fig. 2:**
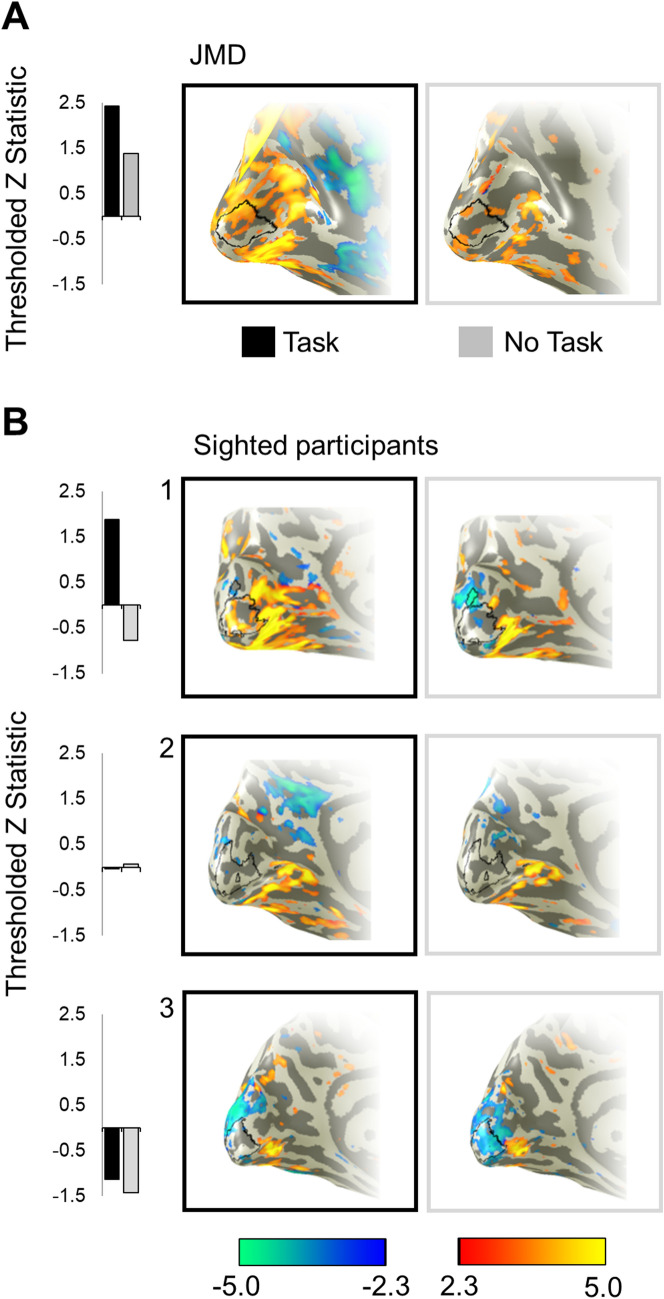
Examples of univariate responses to face stimuli on the inflated cortical surface for JMD (**a**) and 3 control participants (**b**) under two viewing conditions: task, no task. JMD data presented are from the right hemisphere and have been flipped for visualization purposes. Data presented were obtained during adaptation (controls 2 and 3) and no-adaptation (JMD and control 1). Bar graphs illustrate thresholded z statistics to faces under task (black bars) and passive viewing (grey bars) conditions. LPZ represented by black line at the occipital pole

Given that stimuli were presented monocularly and appeared in one hemifield (right for controls, left for JMD), we only report data from the left hemisphere in controls, and the right hemisphere in JMD. This approach is consistent with that used by other researchers (Baker et al. 2005). In Fig. 2, we provide thresholded statistical maps illustrating responses in the LPZ in JMD and three representative controls, to highlight the variability in response patterns across individuals in both adaptation and no-adaptation conditions. When completing the task, JMD exhibited an increase in LPZ responses particularly when viewing faces (Fig. 2A). The positive BOLD responses are not limited to the ROI in which we analyse the data below, but we note that the LPZ represents a visual field area considerably smaller than JMD’s visual field defect. It is not surprising therefore that the positive signals are observed outside of the ROI. Furthermore, positive signals in even more peripheral representations likely arise from the stimulus-related responses, which in their own right can be modulated by task. Whilst a slight increase is also observed when completing a task with scrambled faces, this effect was not as strong. JMD clearly shows a marked increase in BOLD in percent signal change within the LPZ, typically taken as a signature of reorganisation of function. Control 1 behaves in a similar manner to JMD, exhibiting a task-related response to faces (*z* > 2.3, *p* < 0.05). Control 2 shows no marked increase or decrease in LPZ responses across all stimulus and task conditions. Finally, control 3 shows a large amount of negative BOLD for all stimulus and task conditions in terms of amplitude.

**Fig. 3:**
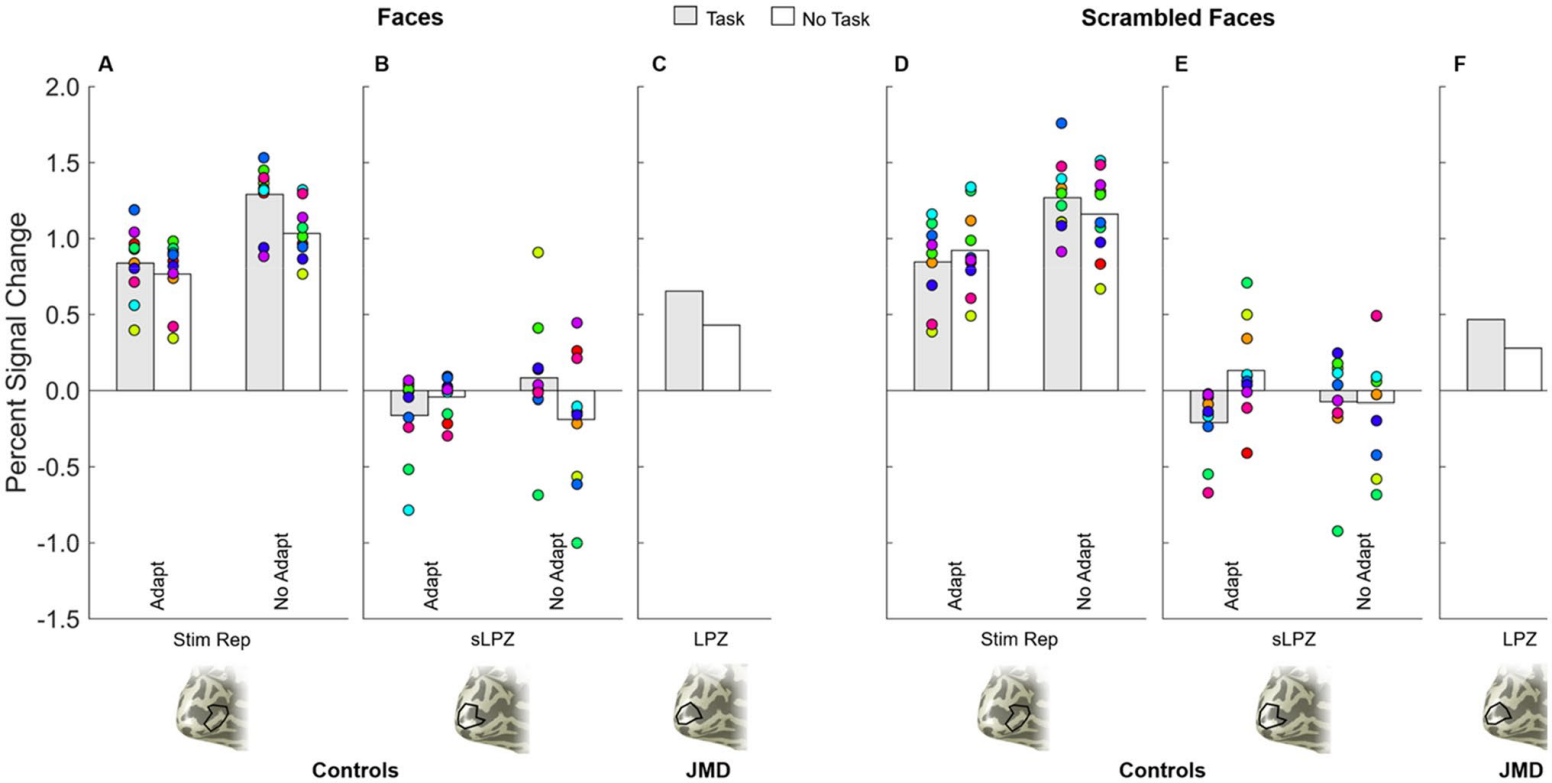
Summary statistics for controls (**a, b, d, e**) and JMD (**c, f**), for faces (**a**–**c**) and scrambled faces (**d–f**) under two viewing conditions: task (shaded bars), no task (open bars). Control group average for adaptation and no-adaptation for each ROI are represented by the bars—stimulus representation (graphs **a** and **d**, lower bank of the calcarine, anterior to the sLPZ) and the simulated LPZ (graphs **b** and **e**). Each individual control is represented by a coloured dot

As patient studies focus on individual data, we plotted the percent signal change for each individual participant (coloured dots in Fig. 3) for both adaptation and no-adaptation experiments. Data for the controls show that all individuals exhibit positive univariate responses in the cortical representation of the stimulus (irrespective of whether the stimuli were intact or scrambled faces—Fig. 3A, D). There is also a hint that a stimulus-related task may enhance these stimulus-driven responses (shaded bars are greater than open bars in three of the four conditions). The individual control responses from the sLPZ are distributed around a mean that is close to zero in all conditions. However, there are individuals that exhibited relatively large positive univariate responses in the sLPZ (Fig. 3B, E). Indeed, some of the control responses are as large as or exceed those found in the LPZ of JMD, who had visual loss. The patient, JMD, exhibits consistent positive responses in the LPZ that also appear to be modulated by the task, but little by the stimulus (Fig. 3C, F).

### Group LPZ BOLD responses

#### LPZ

We analysed the control LPZ responses using a 2 × 2 × 2 (adaptation × task × stimulus) repeated measures ANOVA which confirmed there was no significant main effect of adaptation, *F*(1,9) = 0.003*, p* = 0.957, stimulus type, *F*(1,9) = 0.187, *p* = 0.676 or task condition, *F*(1,9) = 0.328, *p* = 0.581. Therefore, our hypothesis that a more realistic simulation of the retinal lesion would unmask feedback is not supported. We were also unable to detect any effect of task on responses that has previously been observed in the LPZ responses from patients. It is possible that responses could emerge as a result of adapting and performing a task which would be highlighted by an adaptation by task interaction. This two-way interaction was however not significant (*F*(1,9) = 3.67*, p* = 0.088). The results of this analysis are consistent with, but do not explicitly test for an absence of a positive mean univariate response in the sLPZ. To address this, we ran a series of one-sample *t*-tests—one for each condition—to assess whether we observe positive responses significantly above zero. For the sLPZ, there were no significant positive responses for any combination of adaptation, stimulus or task (*t(*9) ranged from −1.80 to 1.38, all *p* > 0.105). One result was significant (adaptation, scrambled faces, task), but this was a negative response in the sLPZ, indicative of an increase in negative BOLD (*t*(9) = −2.958, *p* = 0.016).

#### Stimulus representation

The same ANOVA approach as described above was used to investigate the responses obtained from the stimulus representation in controls. In this case, there was a significant main effect of adaptation on responses, *F*(1,9) = 22.03*, p* = 0.001, but no main effect of stimulus type, *F*(1,9) = 3.38, *p* = 0.099 or task, *F*(1,9) = 2.947, *p* = 0.120. While not reaching significance with an ANOVA, the one-tailed trend for larger responses elicited during a stimulus-related task (*p* = 0.060) and for scrambled images (*p* = 0.050) are consistent with previous research. The plausible two-way interaction between adaptation and task was not significant (*F*(1,9) = 4.00*, p* = 0.076). To be consistent with the approach taken above, we also ran a series of one-sample *t*-tests to determine whether responses in the stimulus representation were significantly different from zero (in the positive direction). As expected, all combinations of adaptation, stimulus type and task emerged as significant in the positive direction (*t(*9) ranged from 10.14 to 19.30, all *p* < 0.001).

### Reliability analysis

**Fig. 4:**
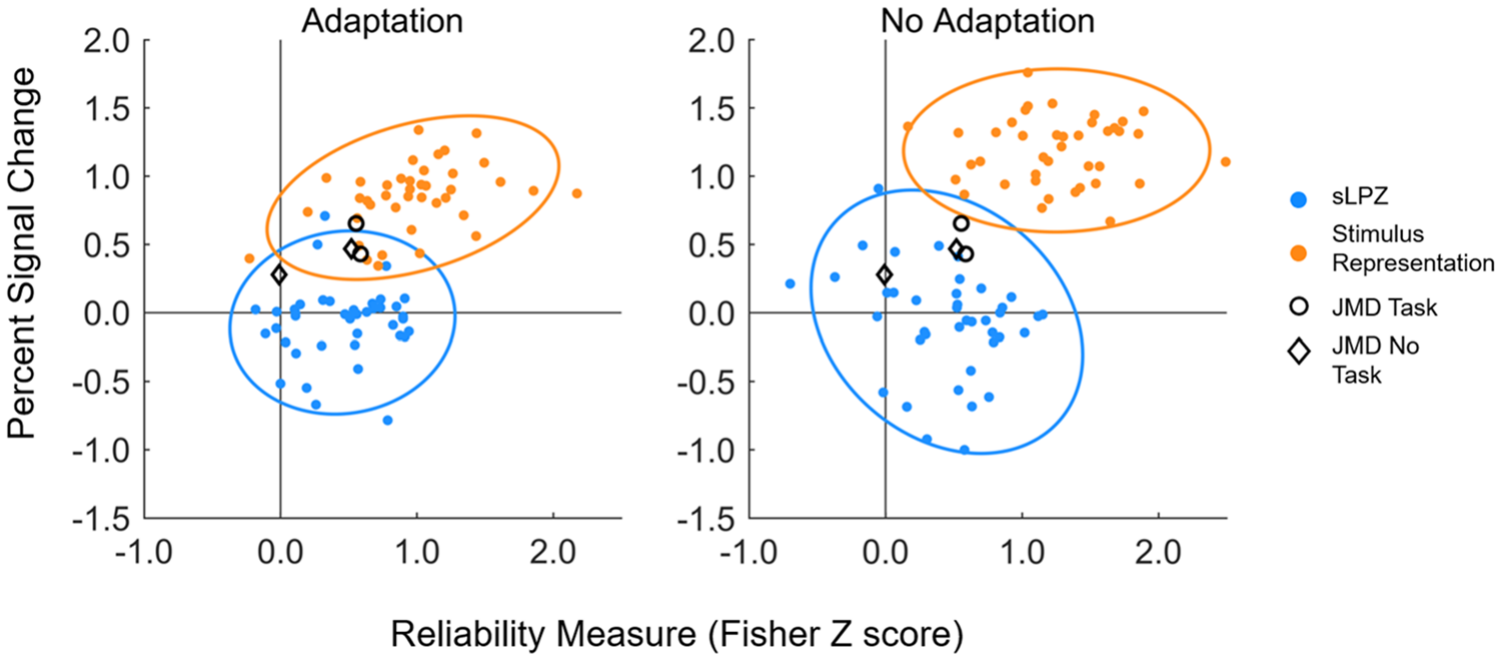
Reliability analysis: scatterplots showing all data for all stimuli and viewing conditions (faces, scrambled faces, task, no task). Data from the bleaching experiment (left) and no bleaching (right) are plotted for both the LPZ (blue) and stimulus representation (orange) ROIs. Reliability measure (transformed to a Fisher *Z* score, with positive numbers indicating more reliable responses) is plotted against the univariate response, measured in percent signal change. Ellipses represent 95% confidence intervals. Despite the difference in the polarity of the responses in the sLPZ, responses seem largely reliable. JMD results (black) lie on the fringe of the data cloud, showing higher reliability and greater responses in the LPZ

Thus far, we have examined the mean of the control group responses, which shows no evidence of being positive. At the same time, however, some individual control participants exhibit responses in the sLPZ that exceed those responses from the JMD patient. To interrogate the reliability of the individual responses from the LPZ, patterns of response across voxels within the LPZ were correlated across experimental runs within each condition, for example, run 1 no task with run 2 no task for faces. The reliability measure (correlation converted to a Fisher *Z* score) was plotted against the percent signal change observed in the univariate analysis (Fig. 4). Results for the sLPZ indicate patterns of responses within individuals were more reliable across runs than expected by chance as indicated by the generally positive Z scores (blue data points in Fig. 4). This contrasts with the distribution of the univariate response across the group being centred on zero (on the vertical axis). The responses from the stimulus representation of controls are both largely reliable and have a positive mean.

Data for JMD lie on the fringe of the data ‘cloud’ consisting of all control data points for the sLPZ (JMD represented by black markers in Fig. 4). Two data points represent task conditions for each stimulus type and two represent passive viewing of the same stimuli. The two most reliable responses are for the stimulus-related task, closely followed by the passive viewing of faces, with passive viewing of scrambled faces appearing least reliable. It is also true that by lying on the fringe of the distribution of control data originating from LPZ responses, JMD’s data also lie near the fringe of the distribution of control data originating from the responses from stimulus representation. In the case of the patient, therefore, it is a challenge to categorise the responses from the LPZ as being consistent with either control responses from the sLPZ or the stimulus representation.

### Classifier analysis

Next, we combined the measures of amplitude and reliability to categorise responses in controls. Using the leave-one-out cross-validation method, the linear discriminant analysis classified responses in the adaptation experiment as coming from the unstimulated zone (LPZ) and stimulus representation with 93.75% accuracy. For the no-adaptation condition, accuracy reached 98.75%. The LDA classifier trained on adaptation data for controls, classified JMD responses as most comparable to the responses originating from the stimulus representation of sighted controls (3 of the 4 data points classified as stimulus representation). However, an LDA trained on no-adaptation data classified the same JMD responses as being most comparable to the unstimulated zone (LPZ) in sighted controls (all 4 data were classified as originating from the sLPZ). This supports data shown in Fig. 4, illustrating that JMD responses fall largely within the 95% confidence intervals for the stimulus representation data in the adaptation condition, and all fall within 95% confidence intervals for the sLPZ for the no-adaptation condition.

## Discussion

Our overall aim was to determine whether an fMRI signature of reorganisation of visual processing is unique to patients with retinal lesions or whether they can be detected in normally sighted individuals. To explore this, we asked whether we could (a) create a more realistic simulation of a retinal lesion that might unmask responses in the simulated LPZ, and (b) assess signals in the sLPZ under different viewing conditions (task vs passive viewing). We predicted that signals would be more likely to emerge during stimulus-related task conditions. Our results suggest that our attempt to create a more realistic simulation of a retinal lesion did not alter responses in the sLPZ. Additionally, whilst at the group level, controls did not mimic the LPZ response of a patient with retinal lesions, an fMRI signature of reorganisation of visual processing can be observed in some, but not all, normally sighted individuals. Given that only a minority of patients display responses in the LPZ too (Morland 2015), careful consideration of evidence for reorganisation from LPZ signals is required.

We examined the control data on an individual basis to be consistent with case / case series studies reported in the literature. Some controls responded in a similar manner to JMD showing task-related modulations of positive responses in the sLPZ. These results are consistent with those reported by Masuda and colleagues, who found some patients exhibited the same task-related modulations of signals from the LPZ for visual, tactile and auditory stimuli (Masuda et al. 2008, 2010, 2021). The JMD patient we tested, therefore, exhibited the hallmark of reorganisation of visual processing that has been detected in many studies, but not all patients (Baker et al. 2005, 2008; Dilks et al. 2014). At the same time, however, the same signature of reorganisation of visual processing could be detected in individual control participants. The number of these controls (22%) falls within the number of patients also showing sLPZ responses in the literature—between 0 and 50% (Morland 2015). Therefore, responses in the LPZ may not be unique to patients. Our reliability analysis revealed that signals from the sLPZ of controls are not random, and that some controls exhibited responses from the sLPZ that were as large and reliable as those found in the patient. Further to this, we analysed our control data as a group which revealed a distribution of the univariate responses that are centred around zero in the sLPZ for both adaptation and no-adaptation conditions, therefore suggesting that responses in the sLPZ observed in some individuals are not a general property of the group on average.

Our second prediction that a stimulus-related task could plausibly enhance signals from the sLPZ, particularly during adaptation conditions was also not supported by our results from controls. Given previous work has focused on case/case series, it is clear that if group averages were computed for the patients assessed by Baker et al. (2005, 2008), Dilks et al. (2009), Masuda et al. (2008), a better assessment of the effect could be achieved. This highlights the need to shift from case and case series approaches to group studies. In an earlier group study, we found no evidence of responses in the LPZ of patients (Baseler et al. 2011), but it is noted that the stimulus and task conditions did not reproduce those used in studies that have detected responses in the LPZ (Baker et al. 2005, 2008; Dilks et al. 2009, 2014). Our study can shed light on what sample sizes would be required to distinguish LPZ signals in patients from those from controls. The positive responses in JMD’s LPZ were 0.654 and 0.468 during the one-back task for face stimuli and their scrambled counterparts, respectively. The standard deviations for the 10 controls varied between 0.103 and 0.323. To be cautious, therefore, we select the higher value of the standard deviation and couple it with the lower value (0.468) of the JMD response. Importantly, the effect we observe in JMD would be significant at *p* < 0.05 for a sample size of 8 if that effect represented the mean of patient responses. However, it is important to note that if the effect was smaller, the sample size would have to increase markedly, for example, if the patient response mean was halved, the sample size would increase to 31.

Our motivation to examine adaptation came from the framework put forward by Masuda et al. (2008) to explain signals in the LPZ of patients. They proposed that feedback was the source of responses in V1 and that in the absence of incoming signals to V1 from the retina, the feedback registered a significant positive response in V1’s LPZ. Further, they reasoned that feedback to V1 would not register as a signal in the sLPZ of controls because the retina would be signalling the presence of a zero contrast within the macula to V1, effectively cancelling out the feedback signals to V1. Our approach therefore was to assess the effect of dissociating macula signals to V1 from signals that would encode the stimulus. We did this by adapting the macula to bright light such that no stimulus contrast, whether it was zero or greater could be relayed to V1. Potentially, this adaptation could unmask, in controls, the feedback signals that V1 receives. Our results however did not highlight an average positive response in the sLPZ during adaptation or during no-adaptation and no significant differences between the signals from each condition emerged. The second aspect of Masuda et al.’s framework is that signals in the LPZ emerge only during a stimulus-related task, again consistent with the feedback hypothesis. We tested therefore whether an interaction with task and adaptation might underpin our results but found it did not.

The lack of a positive shift in the group mean of LPZ signals in the adaptation condition could indicate that the adaptation we used, while a better simulation of retinal lesions, did not match closely enough the total absence of retinal signalling found in patients. If that were the case, the cancellation of feedback signals by signalling along retinal afferents as proposed by Masuda et al. could still take place. Consistent with the presence of retinal signalling after adaptation was the report from participants of a strong visible afterimage with a sharp boundary. Our adaptation approach therefore can be thought of as saturating retinal stimulation rather than removing it. Moreover, the adaptation is relatively brief compared to light deprivation studies that were able to reveal tactile responses in controls following 5 days of blindfold wearing (Merabet et al. 2008). In the context of the Masuda model, therefore, the adaptation may only have served to secure a strong and relatively constant incoming signal to V1, which in turn would prevent the unmasking of feedback signals. It is possible that more lengthy light adaptation or as previously noted, deprivation is the key to unmasking task-dependent feedback signals to V1. Even so, our work contributes to the literature in so far as it shows that another manipulation to the experimental approach results in no detectable change in sLPZ responses at the group level. It is now established therefore that the use of a black (Lerner et al. 2006), uniform grey (Baker et al. 2005, 2008; Baseler et al. 2011; Dilks et al. 2009, 2014; Schumacher et al. 2008; Sunness et al. 2004) or adapted stimulus design generates largely equivalent results.

Finally, we have also shown that individual responses from the LPZ, despite varying in strength and polarity, are reliable. The reliability measure we computed provides some context. Overall, it seems responses were reliable for both adaptation and no-adaptation conditions, suggesting that perhaps the pattern of responses in the sLPZ, rather than the univariate response, contained some stimulus information. This is consistent with previous work reporting patterns of response in the representation of an unstimulated part of the visual field contained contextual information for the scenes presented to the surrounding regions (Smith and Muckli 2010). It should be noted however that in such studies, there is an obvious missing component to the image that participants are presented, which is not the case in our study. Our results are perhaps more like the work of Williams et al (2008), who presented stimuli in peripheral locations and found reliable signals in central representations. The origin of such responses could be larger draining veins that register BOLD responses—and reliable ones (Olman et al. 2007; Turner et al. 1998). However, these responses are not necessarily tied to the location of the neurons that may drive oxygen-level changes in these larger vessels. Using alternative imaging protocols that are less susceptible to large vessel artefacts, such as spin-echo sequences, would shed light on the origin of BOLD response patterns detected in the LPZ (Howseman and Bowtell 1999; Yacoub et al. 2003).

Our approach to use both the univariate response along with the reliability of responses also has the potential to help understand whether a reorganisation of visual processing genuinely occurs in patients. As expected, responses from the stimulus representation in controls are positive and reliable. Unequivocal evidence of reorganisation of visual processing following loss of visual input would register as similarly reliable and positive signals within the LPZ of patients (Morland 2015). Whilst JMD does show reliable, positive signals overall, they do not appear to be entirely different from some responses found in the sLPZ in controls. Indeed, the use of response amplitude and reliability in classifying the LPZ responses of the patient resulted in a greater number of classifications being associated with normal responses from the LPZ rather than the stimulus representation.

Determining the source of LPZ responses is important; if there is evidence of reorganisation of visual processing, this will interfere with the success of visual restoration (Baseler et al. 2011; Morland 2015). Even if the function of the eye was to be restored, it relies on the parts of the visual brain which previously processed input from the macula to remain capable of processing new incoming information. Effectively, cortex would have to reorganise again to resume its former role of processing information from central representation, to allow for some form of functionally useful vision. This clearly is not the desired outcome, and so the reliability of LPZ responses therefore needs to be considered in future work to determine if those responses represent genuine evidence of reorganisation of visual processing.

## Data Availability

Participants consented the use of their data by the investigators and to third parties, but only after the use of the data by those third parties was scrutinized by the York Diagnostic Imaging Centre's Research Governance Committee. The corresponding author may be contacted to initiate data sharing and assistance with seeking ethical approval for the use of the data.
